# *Ureaplasma*-driven inhibition of the epithelial Na^+^ transport in fetal alveolar cells: A novel mechanism of *Ureaplasma*-mediated preterm lung disease

**DOI:** 10.1371/journal.ppat.1013837

**Published:** 2025-12-29

**Authors:** Kirsten Glaser, Carl-Bernd Rieger, Elisabeth Paluszkiewicz, Ulrich H. Thome, Mandy Laube

**Affiliations:** 1 Center for Pediatric Research Leipzig, Division of Neonatology, Department of Pediatrics, University of Leipzig, Leipzig, Germany; 2 Division of Neonatology, Department of Women’s and Children’s Health, University of Leipzig Medical Center, Leipzig, Germany; University of North Carolina at Chapel Hill, UNITED STATES OF AMERICA

## Abstract

Respiratory tract colonization with *Ureaplasma* species has been repeatedly associated with the development of acute and long-term pulmonary morbidity in preterm infants. However, despite strong evidence from observational studies and animal models, apart from inflammation, underlying mechanisms of *Ureaplasma*-driven lung disease, such as potential functional impairments, are mainly unknown. Knowledge of *Ureaplasma*-lung interaction and *Ureaplasma* virulence factors is scarce. The present investigation is the first to examine the influence of perinatal *Ureaplasma* infection on critical mechanisms of alveolar fluid clearance (AFC) in immature lung cells, which drive perinatal transition from fluid-filled lungs before birth to alveolar fluid absorption, enabling lung breathing. Disruption or impairment of these mechanisms could worsen respiratory distress in preterm infants and contribute to acute lung injury. Moreover, the present study addressed *Ureaplasma*-derived ammonia and the accompanying pH shift as potential virulence factors driving *Ureaplasma*-host interactions. Both have long been discussed as virulence factors, but remain unexamined, so far. We report that viable *Ureaplasma* isolates induced a 30–90% decrease in epithelial Na^+^ transport of primary rat fetal distal lung epithelial (FDLE) cells upon 24 hours of infection. Moreover, the decrease was linked to a significant inhibition of the epithelial Na^+^ channel (ENaC) and Na,K-ATPase activities, both mediating the essential AFC. It was observed that acute *Ureaplasma* infection induced phosphorylation of Erk1/2 – a well-known inhibitor of ENaC activity. Notably, exposure of FDLE cells to *Ureaplasma*-driven NH_3_ – in contrast to the hydrolysis-driven pH shift – fully mimicked *Ureaplasma*-driven effects and inhibited the epithelial Na^+^ transport. Co-incubation with the urease inhibitor flurofamide entirely restored Na^+^ transport in *Ureaplasma*-infected FDLE cells. *Ureaplasma* infection differentially modulated ENaC subunit and surfactant protein mRNA expression. In summary, the present study revealed a functional impairment of fetal pulmonary epithelial cells upon acute *Ureaplasma* infection and identified NH_3_ as a *Ureaplasma* virulence factor in this context. Co-incubation with flurofamide restored Na^+^ transport. This study describes a novel mechanism of *Ureaplasma*-driven early preterm lung disease, which might be of great significance for a deeper understanding of *Ureaplasma*-host interactions. Notably, the present findings offer a potential therapeutic role for urease inhibitors in *Ureaplasma*-colonized preterm infants.

## Author summary

Long dismissed as pathogens in preterm infants, *Ureaplasma* species represent a potentially actionable factor causing acute and chronic preterm lung disease, including bronchopulmonary dysplasia (BPD), especially in the most immature infants. Yet, data on *Ureaplasma*-host interactions and *Ureaplasma* virulence factors are scarce, and underlying mechanisms of *Ureaplasma*-associated preterm lung disease remain largely unaddressed. However, against the background of perinatal respiratory tract colonization in 30–60% of very preterm infants, a comprehensive understanding of *Ureaplasma*-driven preterm lung disease remains paramount to identifying addressable pathways and establishing focused therapeutic approaches. Our study is the first to examine the interaction of *Ureaplasma* with fetal distal lung epithelial cells as an established model of otherwise unavailable preterm alveoli and to investigate the influence of perinatal *Ureaplasma* infection on key mechanisms of alveolar fluid clearance (AFC) in the developing lung – an essential event enabling postnatal lung breathing. Our data shed light on a direct crosstalk between *Ureaplasma* and alveolar epithelial cells with potential impairment of the key mechanisms driving AFC. The present study reveals a *Ureaplasma*-mediated affection of alveolar cell function that might significantly impact postnatal transition to air breathing. We report a significant decrease in the epithelial Na^+^ transport in *Ureaplasma*-infected cells resulting from inhibition of epithelial Na^+^ channel (ENaC) and Na,K-ATPase activities, and we describe *Ureaplasma*-mediated NH_3_ as the potential causal virulence factor, offering a potential therapeutic role for urease inhibitors in *Ureaplasma-*colonized preterm infants.

## Introduction

The two *Ureaplasma* species, *Ureaplasma* (*U*.) *parvum* (serovars 1, 3, 6, 14) and *U. urealyticum* (serovars 2, 4, 5, 7–13), are mainly commensal bacteria of the adult urogenital tract. Yet, in preterm infants, consistent epidemiological and experimental data indicate a strong correlation between *Ureaplasma* respiratory tract colonization and adverse pulmonary short- and long-term outcomes, including bronchopulmonary dysplasia (BPD) [1–3]. Colonization rates increase with decreasing gestational age, leaving the most immature preterm infants at the highest risk. Noteworthy, *Ureaplasma* species are detected in early life respiratory tract samples of about 30% of preterm infants born below 30 weeks of gestation and up to 60% of infants born below 26 weeks [3,4]. Animal studies and *in vitro* data confirm inflammatory responses and altered lung development in preterm mice, sheep, rhesus macaques, and baboons exposed to intrauterine *Ureaplasma* infection, as well as in *Ureaplasma*-infected neonatal monocytes [4–6]. However, besides the indirect effects of systemic inflammation and direct apoptosis-modulating effects of *Ureaplasma* on pulmonary epithelial and endothelial cells [7,8], the underlying mechanisms of *Ureaplasma*-driven preterm acute and chronic lung disease are largely unknown. Knowledge of *Ureaplasma*-host interactions and *Ureaplasma* virulence factors is scarce. A surface-exposed family of lipoproteins termed “multiple banded antigen” has been considered the major virulence factor [9]. Hydrolysis-driven pH shift and the accompanying ammonia accumulation have long been discussed as another virulence factor potentially exerting cytotoxic effects, though remaining unexamined, so far [2,9]. Acknowledging that major morbidities, such as BPD, remain high in the most immature preterm infants in times of ever-decreasing gestational age, a better knowledge of *Ureaplasma*-driven preterm lung disease remains paramount to identifying addressable pathways and further developing therapeutic strategies. Notably, current antimicrobial treatment strategies may be effective in *Ureaplasma* eradication in preterm infants but might not prevent *Ureaplasma*-driven adverse neonatal outcomes.

Serovars of both *Ureaplasma* species have been identified in clinical samples from pregnant women and preterm infants. An association between specific species or serovars and adverse pregnancy or neonatal outcomes is not definitively established [9–12]. While some studies reported on a predominance of *U. parvum* [11,13,14], others have not reported any predominance of particular species or serovars [12,15]. In this study, representatives from both species were examined to evaluate potential differences in virulence.

One key mechanism driving the neonate’s adaptation to air breathing is the early postnatal alveolar fluid clearance (AFC). While intrapulmonary fluid is essential for fetal lung development, perinatally, this intrapulmonary fluid must be removed efficiently and quickly to fully establish postnatal lung function. This perinatal lung transition is impaired in preterm infants due to structural and functional lung immaturity [16]. The subsequent respiratory distress syndrome (RDS) is a leading cause of preterm morbidity and mortality [17]. Besides surfactant deficiency, an immature epithelial Na^+^ channel (ENaC) expression has been acknowledged as a critical mechanism [16]. ENaC in the apical membrane compartment of alveolar type II (ATII) cells and the basolateral Na,K-ATPase promote the epithelial Na^+^ transport [18], which osmotically drives fluid absorption from the alveolar lumen into the interstitium and circulation. Decreased AFC in preterm infants [19] is likely due to a lower ENaC expression [20]. Knockout of the α-ENaC subunit led to RDS and respiratory failure in newborn mice [21]. In ATII cells, ENaC comprises three homologous subunits, α-, β-, and γ-ENaC [18,22], while the Na,K-ATPase contains α_1_- and β_1_-subunits [22]. Perinatal impairment of ENaC function and critical AFC mechanisms in *Ureaplasma*-colonized alveoli could worsen respiratory distress in preterm infants and contribute to acute lung injury. The present investigation is the first to examine the potential impact of perinatal *Ureaplasma* infection on critical mechanisms of perinatal lung transition in preterm infants, comprising epithelial Na^+^ transport, barrier integrity, metabolic activity, gene expression, and kinase signaling. We hypothesized that perinatal alveolar *Ureaplasma* colonization might interfere with early-life lung function and contribute to acute lung injury in colonized preterm infants by mechanisms other than inflammation.

## Results

### *Ureaplasma* infection inhibited Na^+^ transport in fetal alveolar cells

**Fig 1:**
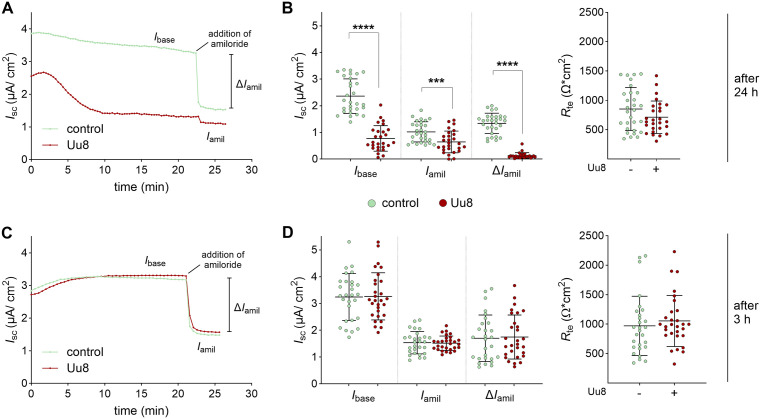
*Ureaplasma* infection strongly reduced epithelial Na^+^ transport in FDLE cells at 24 h incubation. FDLE cells were infected with serovar 8 of *U. urealyticum* (Uu8) for 24 h (A, B) or 3 h (C, D) before analyses. **(A)** Representative Ussing chamber current tracing of Uu8-infected FDLE cells versus controls after 24 h. **(B)** Uu8 infection significantly decreased Na^+^ transport and ENaC activity (*I*_base_, *I*_amil_, and Δ*I*_amil_). The barrier integrity (*R*_te_) of infected FDLE cells (n = 29) was not significantly altered compared to uninfected controls (n = 27). **(C)** Representative current tracing of Uu8-infected FDLE cells versus controls after 3 h. **(D)** Infection of FDLE cells with Uu8 for 3 h did not affect *I*_base_, *I*_amil_, Δ*I*_amil_ or *R*_te_ (n = 29) compared to control cells (n = 28). Statistical analysis: ***p < 0.001, ****p < 0.0001 by unpaired t-test.

A 24-h infection of FDLE cells with *U. urealyticum* serovar 8 (Uu8) reduced the epithelial Na^+^ transport by 70–90%. Ussing chamber analyses demonstrated that Na^+^ transport (basal *I*_SC_, *I*_base_) was significantly decreased by Uu8 infection compared with uninfected cells (Fig 1B). ENaC activity (amiloride-sensitive ∆*I*_SC_, Δ*I*_amil_) was also significantly reduced by Uu8 infection by more than 90%. In contrast, the epithelial barrier function, assessed as transepithelial resistance (*R*_te_), was unaffected. Furthermore, the amiloride-insensitive *I*_SC_ (*I*_amil_), most likely Cl^-^ transport, was significantly reduced by Uu8 (Fig 1B). In contrast, 3 h incubation did not affect Na^+^ transport (Fig 1D).

**Fig 2:**
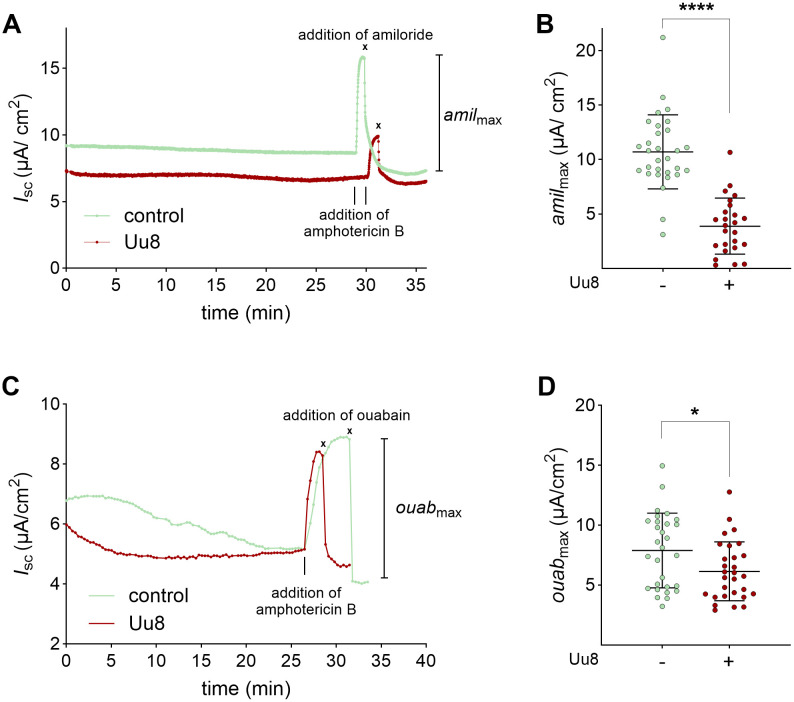
*Ureaplasma* infection reduced isolated maximal ENaC and Na,K-ATPase activities. FDLE cells were infected with Uu8 for 24 h. The maximal activity of each Na^+^ transporter was determined separately by permeabilizing the opposite membrane. **(A)** Representative current tracings of basolaterally permeabilized Uu8-infected or control cells, measured with a 145:5 apical-to-basolateral Na^+^ gradient. **(B)** Uu8 infection significantly reduced the maximum apical Na^+^ permeability mediated by ENaC (*amil*_max_) (n = 30) compared to controls (n = 25). **(C)** Representative current tracings of apically permeabilized Uu8-infected or control cells. **(D)** Maximum Na,K-ATPase activity (*ouab*_max_) was significantly reduced by Uu8 infection (n = 29) compared to uninfected FDLE cells (n = 30). Statistical analysis: *p < 0.05, ****p < 0.0001 by unpaired t-test.

To consider the capacities of ENaC and the Na,K-ATPase separately, the opposite membrane was permeabilized. The maximum amiloride-sensitive apical membrane permeability (*amil*_max_) was significantly decreased by Uu8 to approximately 35% (Fig 2B). In addition, the maximum ouabain-sensitive Na,K-ATPase activity (*ouab*_max_) was significantly reduced upon Uu8 infection, although not to the same extent (Fig 2D). In summary, Uu8 diminished the apical ENaC activity by more than 60% and the basolateral Na,K-ATPase activity by approximately 20% in FDLE cells upon 24 h infection.

**Fig 3:**
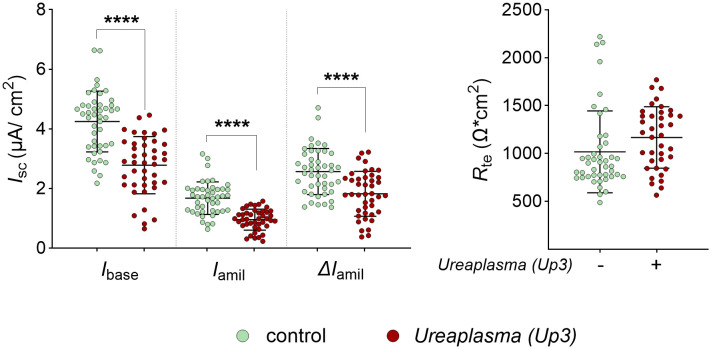
Parallel infection with a *Ureaplasma parvum* serovar 3 reproduced the observed effects on the epithelial Na^+^ transport. Infection with serovar 3 of *U. parvum* (Up3) also reduced epithelial Na^+^ transport in FDLE cells. Cells were infected with Up3 for 24 h before analyses. In line with Uu8 infection, Up3 infection significantly decreased Na^+^ transport and ENaC activity (*I*_base_, *I*_amil_, and Δ*I*_amil_), while *R*_te_ was unaffected (Up3: n = 45; control: n = 42). Statistical analysis: ****p < 0.0001 by unpaired t-test.

To determine if the effects observed for Uu8 were serovar-specific, the electrophysiological measurements were repeated with serovar 3 of *U. parvum* (Up3). Under the same culture conditions, Up3 showed less growth in the same period, resulting in lower pre-infection titers than those obtained for Uu8. However, as seen before, Up3 significantly decreased epithelial Na^+^ transport and ENaC activity in infected FDLE cells compared to uninfected controls (Fig 3). Compared to Uu8, the reduction of Na^+^ transport by Up3 was less pronounced, attenuating ENaC activity by approximately 30%.

### *Ureaplasma* infection induced phosphorylation of Erk1/2

**Fig 4:**
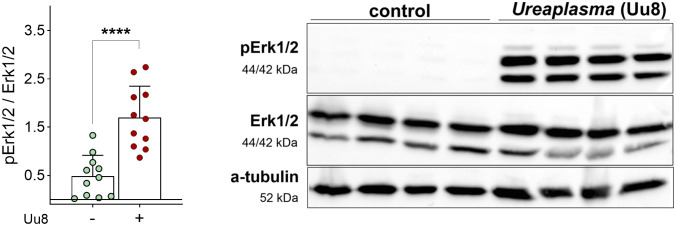
*Ureaplasma* infection significantly activated Erk1/2 signaling in FDLE cells. Erk1/2 phosphorylation was significantly induced upon Uu8 infection (n = 11) compared to controls (n = 11). Representative Western blots give pErk1/2, total Erk1/2 and α-tubulin as loading control. Statistical analysis: ****p < 0.0001 by unpaired t-test.

To investigate whether signaling pathways were disturbed by *Ureaplasma* infection, the phosphorylation of p44/42 MAPK (Erk1/2) was analyzed by Western blot. Erk1/2 is a well-known inhibitor of ENaC function. As given in Fig 4, Uu8 infection strongly increased phosphorylation of Erk1/2 in FDLE cells by more than 3-fold (Figs 4 and S1), possibly contributing to the diminished ENaC activity.

### *Ureaplasma* infection impaired metabolic activity and modulated gene expression in FDLE cells

**Fig 5:**
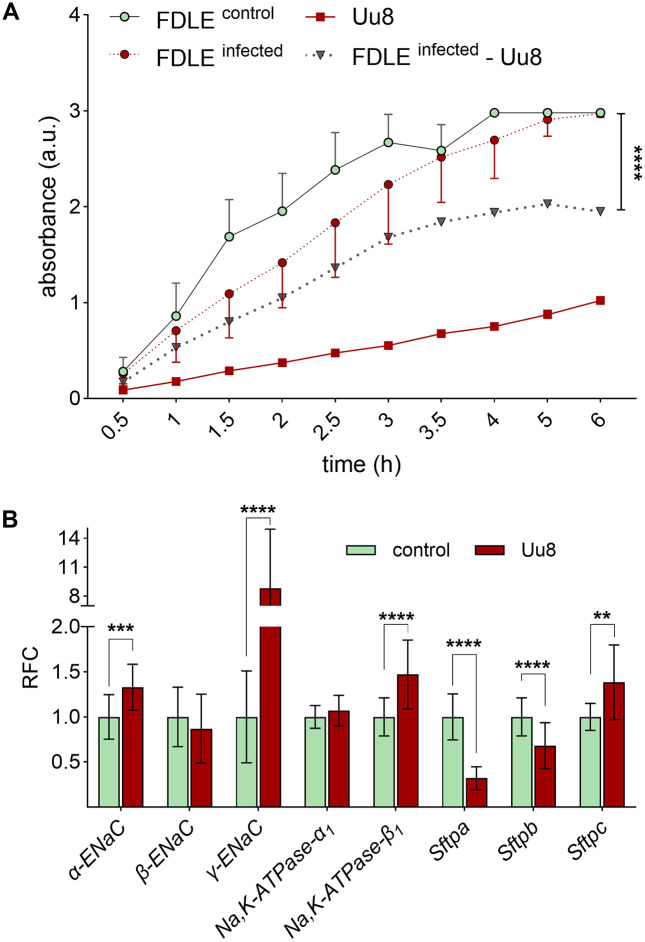
*Ureaplasma* infection significantly affected the metabolic activity and mRNA expression of FDLE cells. **(A)** Metabolic activity was measured every 30 min to 1 h in Uu8-infected FDLE cells, uninfected controls and viable Uu8 isolates. The dotted line represents the metabolic activity of Uu8-infected FDLE cells subtracted by the metabolic activity exhibited by Uu8 themselves*.* Based on this calculation, the metabolic activity was significantly lower in Uu8-infected FDLE cells than in uninfected controls (n = 8-12 per group and time point). **(B)** Uu8 infection significantly increased mRNA expression of *α-* and *γ-ENaC*, *Na,K-ATPase-β*_*1*_, and *Sftpc*. In contrast, Uu8 infection downregulated *Sftpa* and *Sftpb* mRNA expression (n = 12-18). Relative fold change (RFC). Statistical analysis: ****p < 0.0001 by repeated measures two-way ANOVA with Geisser-Greenhouse correction (A) or **p < 0.01, ***p < 0.001, ****p < 0.0001 by unpaired t-test (B).

The metabolic activity was determined by the Water-Soluble Tetrazolium 1 (WST-1) assay in Uu8-infected FDLE cells, uninfected controls, and viable Uu8 isolates. Comparing Uu8-infected FDLE cells with uninfected controls, no significant difference was observed (Fig 5A). However, viable *Ureaplasma* themselves reduced tetrazolium salts even in the absence of FDLE cells. We therefore subtracted the results obtained for Uu8 from those obtained for Uu8-infected FDLE cells and found a significantly impaired metabolic activity in FDLE cells upon infection (Fig 5A).

Surprisingly, Uu8 infection significantly increased mRNA expression of the subunits *α-ENaC* by approximately 30% and *γ-ENaC* by almost 9-fold, while *β-ENaC* mRNA expression was unaffected (Fig 5B). Moreover, Uu8 infection significantly increased mRNA expression of the *Na,K-ATPase-β*_*1*_-subunit by nearly 50%, while the *α*_*1*_-subunit level was not altered. In contrast, the surfactant protein A (*Sftpa*)‘s mRNA expression decreased by approximately 70% and the expression of *Sftpb* by around 30% (Fig 5B). The opposite effect was observed for the *Sftpc* mRNA, increasing by almost 40%. Taken together, the Uu8 infection of FDLE cells increased the mRNA expression of several Na^+^ transporter subunits, in contrast to the observed functional impairment of the epithelial Na^+^ transport.

### Ammonia exposure and not pH shift mimicked *Ureaplasma*-driven effects

**Fig 6:**
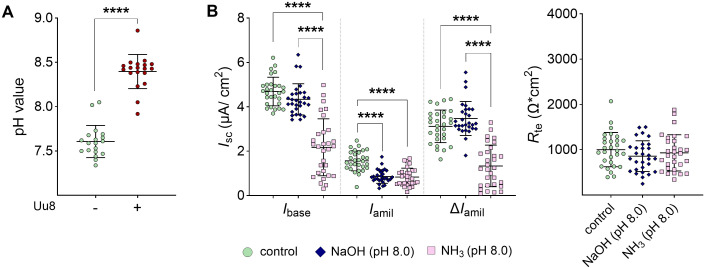
*Ureaplasma*-driven NH_3_ mimicked the effects of acute infection on the epithelial Na^+^ transport. *Ureaplasma* infection shifted the pH in infected FDLE cells due to NH_3_ production. This NH_3_ accumulation mimicked the impact of *Ureaplasma* infection to a large extent. **(A)** Uu8 significantly increased the pH of the FDLE cell medium after 24 h (n = 19 each). **(B)** The pH of the FDLE cell medium was shifted to 8.0 by adding either NaOH or NH_3_ and maintained for 24 h. Exposure of FDLE cells to NH_3_ (n = 30) significantly decreased Na^+^ transport and ENaC activity (*I*_base_, *I*_amil_, and Δ*I*_amil_) compared to control cells (n = 29), while NaOH-driven pH shift (n = 29) only affected *I*_amil_. *R*_te_ was neither affected by NaOH-driven pH shift nor NH_3_. Statistical analysis: ****p < 0.0001 by unpaired t-test (A) or one-way ANOVA with Tukey’s multiple comparison test (B).

A 24-h infection of FDLE cells with *Ureaplasma* caused a relevant pH shift (Fig 6A) due to *Ureaplasma*-driven hydrolysis of urea to ammonia (NH_3_) and the subsequent alkalization of the culture medium. We tested whether the pH shift or NH_3_ was responsible for the observed effects in *Ureaplasma*-infected FDLE cells. The pH shift to 8.0 using NaOH did not affect Na^+^ transport or ENaC activity, whereas adding NH_3_ to FDLE cells strongly reduced the Na^+^ transport (Fig 6B). Taken together, *Ureaplasma*-induced NH_3_ and not the accompanying pH shift inhibited Na^+^ transport and ENaC activity.

**Fig 7:**
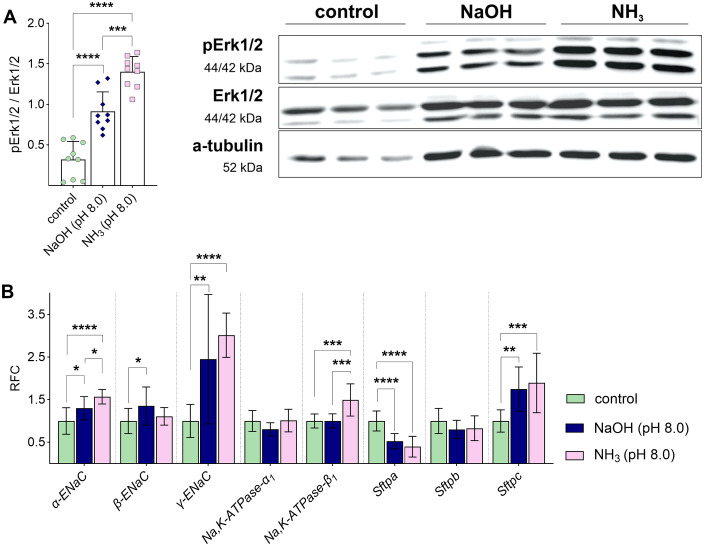
NaOH- and NH_3_-driven pH shift significantly affected Erk1/2 signaling and modulated mRNA expression. FDLE cells were incubated with media containing NaOH or NH_3_ at pH 8.0 for 24 h before analyses. **(A)** NaOH- and NH_3_-driven pH shift to 8.0 significantly increased Erk1/2 phosphorylation (n = 9 each). Representative Western blots of pErk1/2, total Erk1/2 and α-tubulin are given. **(B)** NaOH- and NH_3_-driven pH shift to 8.0 significantly increased mRNA expression of *α-* and *γ-ENaC*, *Na,K-ATPase-β*_*1*_, and *Sftpc*. In contrast, NaOH and NH_3_ downregulated *Sftpa* mRNA expression (n = 11-12). Statistical analysis: *p < 0.05, **p < 0.01, ***p < 0.001, ****p < 0.0001 by one-way ANOVA with Tukey’s multiple comparison test.

Moreover, phosphorylation of Erk1/2 was increased by NaOH-driven pH shift and NH_3_, but the effects of NH_3_ exposure were significantly more pronounced (Figs 7A and S2). In contrast, the *Ureaplasma*-driven increase of α-*ENaC, γ-ENaC,* and *Sftpc* mRNA expression was mimicked by both the NaOH-driven pH shift and the exposure to NH_3_ (Fig 7B). In addition, the NaOH-driven pH shift and NH_3_ exposure both downregulated *Sftpa* mRNA expression (Fig 7B).

### Flurofamide prevents *Ureaplasma*-driven impairment of Na^+^ transport and Erk1/2 phosphorylation

**Fig 8:**
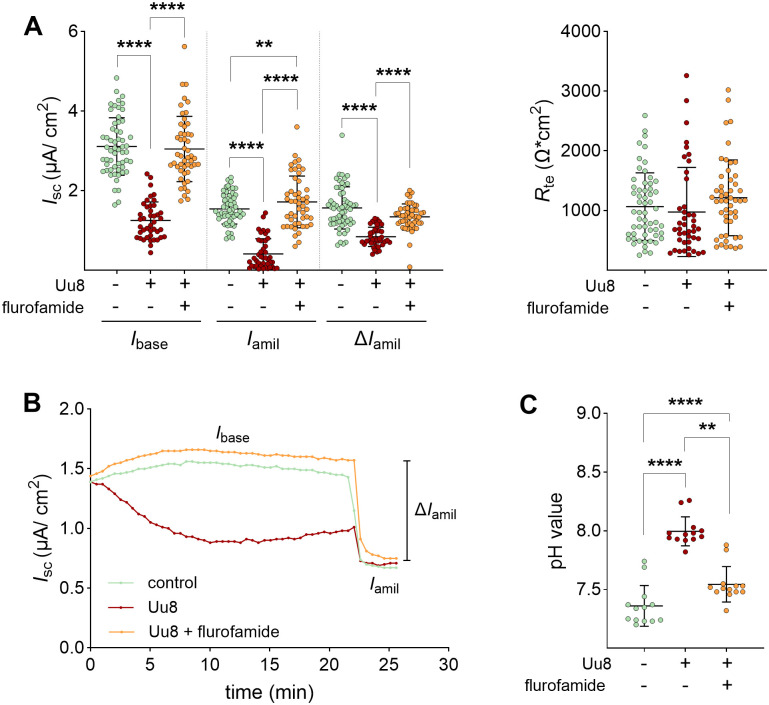
The presence of flurofamide fully restored the *Ureaplasma*-driven reduction of epithelial Na^+^ transport. FDLE cells were infected with Uu8 in the presence or absence of flurofamide (10 µM) for 24 h. **(A)** Uu8 infection (n = 42) significantly decreased Na^+^ transport and ENaC activity (*I*_base_, *I*_amil_, and Δ*I*_amil_) compared to uninfected controls (n = 57). These findings were entirely restored by co-incubating FDLE cells with flurofamide (n = 49). *R*_te_ was neither affected by Uu8 infection nor flurofamide. **(B)** Representative Ussing chamber current tracing of FDLE cells. **(C)** Flurofamide significantly downregulated media pH in Uu8-infected FDLE cells (n = 13 each). Statistical analysis: **p < 0.01, ****p < 0.0001 by one-way ANOVA with Tukey’s multiple comparison test.

Flurofamide is a known inhibitor of bacterial urease. According to the findings above, we hypothesized a role for the *Ureaplasma*-specific urease as a key virulence factor in our experimental setting that might be affected by flurofamide. Thus, FDLE cells were co-incubated with Uu8 and the urease inhibitor to test its ability to restore the Na^+^ transport. Uu8-driven inhibition of the Na^+^ transport was entirely prevented by flurofamide (Fig 8A). Notably, flurofamide ameliorated ENaC activity in Uu8-infected cells to control levels. In addition, flurofamide attenuated the pH shift in Uu8-infected cells (Fig 8C).

**Fig 9:**
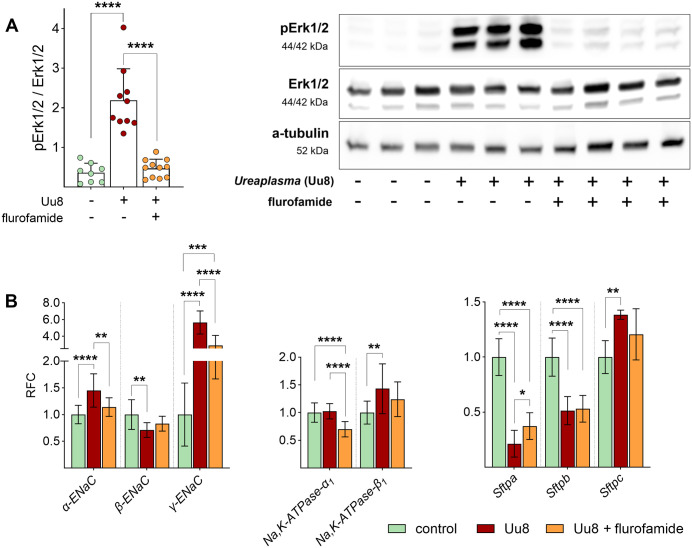
Co-incubation with the urease inhibitor flurofamide prevented *Ureaplasma*-induced phosphorylation of Erk1/2. FDLE cells were infected with Uu8 in the presence or absence of flurofamide (10 µM) for 24 h. **(A)** Addition of flurofamide prevented the Uu8-driven Erk1/2 phosphorylation. Uu8 infection significantly increased Erk1/2 phosphorylation, while elevated phosphorylation was not seen in the presence of flurofamide (n = 8 each). Representative Western blots of pErk1/2, total Erk1/2 and α-tubulin are given. **(B)** Flurofamide had a marginal impact on Uu8-induced changes in mRNA expression of Na^+^ transporters and surfactant proteins. While Uu8 infection significantly increased mRNA expression of *α-* and *γ-ENaC*, *Na,K-ATPase-β*_*1*_, and *Sftpc*, flurofamide diminished the Uu8-induced increase of *α-* and *γ-ENaC* and *Sftpc*. Uu8 infection downregulated mRNA expression of *Sftpa* and *Sftpb,* which was not affected by flurofamide (n = 12 each). Statistical analysis: *p < 0.05, **p < 0.01, ***p < 0.001, ****p < 0.0001 by one-way ANOVA with Tukey’s multiple comparison test.

Notably, flurofamide prevented the Uu8-induced phosphorylation of Erk1/2 (Figs 9A and S3), possibly contributing to the restored ENaC activity. Finally, the Uu8*-*induced increase of *α-ENaC* and *γ-ENaC* mRNA expression was significantly reduced by flurofamide (Fig 9B). *Sftpa* mRNA expression was marginally elevated by flurofamide in Uu8*-*infected FDLE cells.

## Discussion

To our knowledge, this is the first study to examine potential direct effects of *Ureaplasma* species on fetal alveoli and to describe a profound impairment of the epithelial Na^+^ transport in fetal alveolar cells upon acute *Ureaplasma* infection. Respiratory tract colonization of preterm infants with *Ureaplasma* has been repeatedly associated with adverse pulmonary outcome, especially BPD [1–3]. In contrast to the adult respiratory tract, the alveoli of preterm infants – filled with urea-enriched amniotic fluid until the first hours of life – provide a unique and highly favorable environment for *Ureaplasma* species. Since the epithelial Na^+^ transport is essentially involved in perinatal lung transition to air breathing, we sought to determine the potential impact of a perinatal *Ureaplasma* infection on Na^+^ transport-associated mechanisms of AFC.

In FDLE cells, an established model of otherwise unavailable preterm alveoli, we could separate the effects on basolateral Na,K-ATPase, and apical Na^+^ channels by selective permeabilization and observed a significant inhibition of the apical ENaC activity and a less profound reduction of the Na,K-ATPase activity induced by *Ureaplasma* species. While this robust ENaC inhibition emerged upon 24-hour infection, no effect was observed at 3 hours, which is in accordance with *in vitro* studies in other models reporting epithelial injury and inflammatory signaling evident by ~24 hours [8,23], although *Ureaplasma* seem to adhere within a few hours [24]. Notably, diminished epithelial Na^+^ transport in preterm infants [19] has been shown to contribute to the development of RDS [16]. According to our findings, functional impairment of the Na^+^ transport in *Ureaplasma*-colonized infants could further aggravate the critical early-life respiratory situation and worsen the respiratory short- and long-term outcome.

Since 2002, an adjusted taxonomy discriminates the two human species *U. parvum* and *U. urealyticum*. Notably, serovars of both species are found in specimens of pregnant women and preterm infants, and controversy remains whether one of the two species or individual serovars are more frequently associated with diseases than others [9,12]. To investigate potential species-specific pathogenicity, representatives of both species were used in the present study. We confirmed that both the *U. parvum* and the *U. urealyticum* serovar profoundly affected the epithelial Na^+^ transport. However, the *U. urealyticum* serovar exhibited a more potent inhibition, reducing the ENaC activity by 90%, while the *U. parvum* serovar achieved a lesser reduction of about 30%. This less profound effect might not correlate with a generally smaller effect on fetal alveolar cell function but is most likely due to lower titers of Up3 in our experiments. However, overall, the range of *Ureaplasma* inocula used in this study corresponded to concentrations *in vivo* with a *Ureaplasma* load reported in the cervical fluid of pregnancies complicated by preterm labor ranging from 5.0x10^6^ - 1.5x10^8^ cn/ml and the *Ureaplasma* load determined in the amniotic fluid of preterm births ranging from 4.0x10^3^ – 5.2x10^7^ cn/ml [25,26].

Western blot analyses revealed *Ureaplasma*-induced phosphorylation of Erk1/2, a signaling molecule significantly involved in regulating ENaC activity in previous studies. Acknowledging that phosphorylation of Erk1/2 has been shown to diminish ENaC activity in renal cells [27], we speculate that *Ureaplasma*-driven Erk1/2 phosphorylation represents one possible mechanism contributing to the observed ENaC inhibition. This is consistent with published findings in other epithelial systems: ERK activation decreases ENaC open probability without altering surface abundance, resulting in functional inhibition [28,29]. Additionally, ERK activity can promote NEDD4L (neural precursor cell expressed, developmentally downregulated 4)-mediated ubiquitination and degradation of ENaC subunits, thereby reducing channel surface density [30,31]. Importantly, both mechanisms converge on reduced amiloride-sensitive Na⁺ transport, which we directly demonstrate.

WST-1 assays demonstrated a significantly lower metabolic activity in *Ureaplasma*-infected cells that might indicate augmented cytotoxicity. These findings align with the *Ureaplasma*-driven pro-apoptotic and apoptosis-modulating effects described in pulmonary epithelial and endothelial cells [7,8]. In our WST-1 assays, we demonstrated that *Ureaplasma* isolates themselves reduced tetrazolium salts to formazan even in the absence of host cells. Subsequently, the *Ureaplasma*-inherent metabolic activity was subtracted from the results assessed in *Ureaplasma*-infected FDLE cells. Our findings align with genomic sequence analysis*,* suggesting that NADH, reducing the tetrazolium salt WST-1 in the given assay, contributes to several enzyme reactions in *Ureaplasma* metabolic pathways [32].

It is worth noting that the reduced activity of both Na^+^ transporters in FDLE cells was not due to diminished mRNA expression. In fact, the mRNA expression of the *α-* and *γ-ENaC* subunits was increased, possibly reflecting a positive feedback mechanism. The functional relevance of each subunit might well explain the observed expression patterns of the ENaC subunits. In the fetal ovine lung, the *α-* and *γ-ENaC* subunit mRNA expression peaked during labor [33], indicating that these ENaC subunits are critical at the developmental stage of the FDLE cells. On the other hand, overexpression of *β-ENaC* in mice decreases mucus clearance and reduces fluid in the postnatal lung, resulting in a cystic fibrosis-like phenotype [34]. The absent response of *β-ENaC* mRNA expression in FDLE cells in the present study might be explained by its relevance mainly for postnatal lung function.

Notably, this study showed an increased mRNA expression of the *Na,K-ATPase-β*_*1*_ subunit in FDLE cells upon *Ureaplasma* infection. Overexpression of the *β*_*1*_-subunit was previously demonstrated to increase vectorial Na^+^ transport [35], with this subunit being the rate-limiting component in the assembly of the Na,K-ATPase [36]. In the present study, one may speculate that an upregulation of *Na,K-ATPase-β*_*1*_ mRNA expression counteracts *Ureaplasma*-driven diminished Na^+^ transport.

One potential virulence factor of *Ureaplasma* often discussed in the context of *Ureaplasma*-host cell interaction is the urease-mediated generation of NH_3_ and the accompanying pH shift [2,9]. Although frequently considered, these potential virulence factors have not been investigated so far. The present study is the first to describe *Ureaplasma*-derived NH_3_ as a *Ureaplasma* virulence factor in a host-cell specific context affecting the epithelial Na^+^ transport fetal alveolar cells. While a NaOH-mediated pH shift did not affect ENaC activity, exposure of FDLE cells to NH_3_ significantly inhibited the epithelial Na^+^ transport, mimicking *Ureaplasma*-driven effects. These findings demonstrate that the observed effects cannot be attributed to alkalinization *per se*, but require the presence of NH_3_, representing the biologically active species mediating the observed effects. Moreover, NH_3_ exposure also affected mRNA expression of Na^+^ transporters and surfactant proteins and induced Erk1/2 phosphorylation, as observed for the incubation of FDLE cells with viable *Ureaplasma* isolates*.* Notably, a previous report identified two ammonia-specific upregulated phosphorylation sites on Thr202 and Tyr204, known to be phosphorylated by MAP2K1/2, causing activation of ERK1 [37]. In this study using MCF-7 breast cancer cells, quantitative phospho-proteomics strongly suggested that ERK is activated in response to ammonia treatment. Another study supported this finding, showing increased ERK1/2 phosphorylation in response to ammonium and ammonia in MCF-7 cells [38]. Notably, urease-driven virulence has been well described in other urease-expressing pathogens, such as *Proteus mirabilis* [39,40], contributing to epithelial dysfunction and tissue injury, for instance, in urinary tract infections [40,41].

To further delineate the relationship between Na^+^ transport and *Ureaplasma*-driven NH_3_ production, we inhibited the responsible *Ureaplasma* enzyme urease with flurofamide, an urease inhibitor approved by the FDA (*Food and Drug Administration*). Following our hypothesis, flurofamide completely restored Na^+^ transport and ENaC activity in *Ureaplasma*-infected FDLE cells. In addition, flurofamide prevented the *Ureaplasma*-driven phosphorylation of Erk1/2. This aligns with studies confirming that flurofamide inhibits NH_3_ production from urea by intestinal microorganisms *in vitro* [42]. Notably, *Ureaplasma*-driven NH_3_ has been implicated in the pathogenesis of hyperammonemia syndrome in infected immunocompromised patients [43,44]. A mouse model confirmed fatal *Ureaplasma-*induced hyperammonemia in the context of immunosuppression [45] and described reduced blood ammonia levels upon flurofamide treatment [46]. In line with these data, our findings suggest that flurofamide or other urease-inhibiting agents might be promising candidates for future non-antibiotic treatment strategies in *Ureaplasma*-colonized preterm infants. This is especially important since no treatment is available to improve Na^+^ transport and AFC during perinatal lung transition. Moreover, current antimicrobial treatment strategies, mainly using macrolide antibiotics, have several potential side effects and may not prevent long-term adverse neonatal outcomes, according to most recent data [47].

Finally, the gene expression of surfactant proteins was modulated differently in *Ureaplasma*-infected FDLE cells in the present study. *Ureaplasma*-induced downregulation was observed for *Sftpa* and *Sftpb* mRNA expression, while *Sftpc* expression was increased. SP-A contributes to the innate immune system by opsonization, promoting the uptake of various microorganisms by phagocytic cells [48]. Moreover, SP-A protects the alveolar region from uncontrolled inflammatory responses by reducing proinflammatory Toll-like receptor signaling [48]. We speculate that the observed *Ureaplasma*-induced reduction of *Sftpa* expression in our study could possibly weaken alveolar defense. Notably, the present study is the very first investigating a potential impact of *Ureaplasma* respiratory tract infection on alveolar surfactant synthesis, and this mechanism has not been described before. Of interest, *Ureaplasma*-driven downregulation of *Sftpa* might also impair the host’s immune defense against *Ureaplasma*. SP-A was demonstrated to enhance ureaplasmacidal activity in murine alveolar macrophages [49]. SP-B and SP-C are essential for the surface-spreading properties of pulmonary surfactant. Still, only the lack of SP-B is incompatible with life, while SP-C is supposed to contribute to stabilization of surfactant molecule effects on the alveolar structure [48]. In the present study, *Ureaplasma* infection downregulated the indispensable expression of *Sftpb,* potentially further contributing to preterm lung disease. It is worth mentioning that the present data are in line with investigations on lipopolysaccharide (LPS)-induced lung inflammation and lung injury. The latter demonstrated that LPS-induced lung dysfunction corresponds closely to abnormal surfactant function and reduced alveolar *Sftpb* mRNA and SP-B protein expression [50].

**Fig 10:**
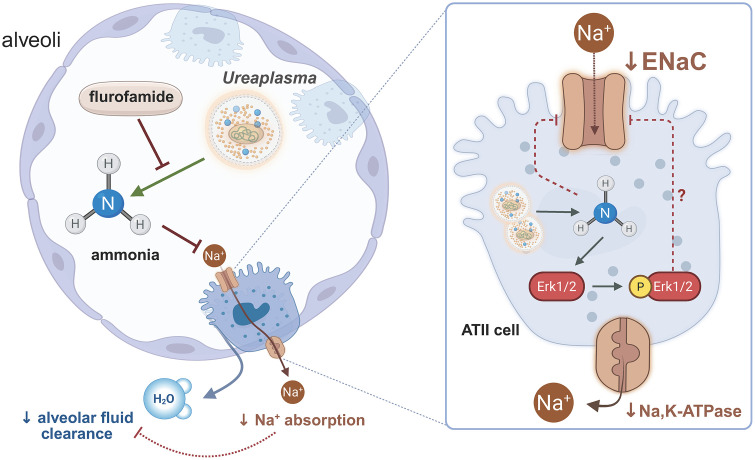
Summarized suggested mechanism of *Ureaplasma*-driven inhibition of Na^+^ transport, impairment of perinatal AFC and transition to air breathing. Our data demonstrate a *Ureaplasma*-driven inhibition of Na^+^ transport in fetal alveolar cells. Moreover, the *Ureaplasma-*driven ammonia production induces Erk1/2 phosphorylation. This might be an underlying mechanism of the observed inhibition of ENaC activity and Na^+^ transport in *Ureaplasma*-infected alveolar epithelial cells. Notably, flurofamide, an inhibitor of the *Ureaplasma*-specific enzyme urease, restores Na^+^ transport and normalizes Erk1/2 phosphorylation in *Ureaplasma*-infected FDLE cells. Created in BioRender. Laube, M. (2025) https://BioRender.com/x30p361.

In summary, this is the very first investigation to determine potential direct interactions of *Ureaplasma* with immature alveoli and to reveal significant functional impairments of alveolar epithelial cells upon acute *Ureaplasma* infection. The present study suggests a global inhibition of the epithelial Na^+^ transport in fetal alveolar cells (Fig 10), potentially due to altered Erk1/2 signaling. *Ureaplasma*-driven accumulation of NH_3_ was identified as a key contributing virulence factor, mimicking most *Ureaplasma-*driven effects on FDLE cell function. In line with this observation, the urease inhibitor flurofamide completely restored Na^+^ transport, ENaC activity, and Erk1/2 activity, confirming the role of the *Ureaplasma*-driven hydrolysis of urea as virulence factor in this context.

Some limitations of this study ought to be acknowledged. We used primary fetal alveolar epithelial cells of rat origin since primary or immortalized human fetal or neonatal cells originating from the deeper respiratory tract of immature preterm infants are unavailable. The given model was the best approximation for the very first study on potential functional impairments upon preterm *Ureaplasma* respiratory tract infection, especially since fetal rat lungs at the saccular stage used in the present study exhibit features of immaturity that are very similar to the underdeveloped lung structures of human preterm infants. Moreover, in the present study, FDLE cells were cultured under medium-submerged conditions that resemble the fetal intrauterine environment, where the developing lung is filled with urea-rich fluid, providing an optimal niche for *Ureaplasma* survival and proliferation. However, the current findings need to be confirmed in other models. In particular, this study ought to promote investigations in a preterm animal model of *Ureaplasma* respiratory tract infection that allows assessment of respiratory function and testing of potential effects of intra-alveolar application of the urease inhibitor flurofamide in *Ureaplasma*-infected animals. Further research in the given *in vitro* setting will include the incorporation of neonatal immune cells, mimicking the presence of alveolar macrophages, and will investigate the effect of other bacteria potentially present perinatally in the preterm alveoli but not generating NH_3_ as *Ureaplasma* species do. The two existing *Ureaplasma* species were both tested in the present study. However, we acknowledge that pre-selecting specific serovars may have caused selection bias.

Finally, we would like to clarify that Western blot analysis of ENaC subunits was not included in this study since reliable detection of ENaC proteins in lung tissue or primary alveolar epithelial cells is technically challenging, especially in the fetal state, where ENaC expression is shifting as the lung transitions from fluid secretion in utero to fluid absorption at birth [51,52]. In contrast to kidney or colon epithelia, ENaC expression in the lung is relatively low and developmentally regulated, often resulting in weak and variable bands that are hardly quantitatively interpretable. This limitation has been well documented in previous studies (e.g., [29,53–55]. Therefore, we focused on functional measurements of amiloride-sensitive Na⁺ transport, which directly reflect ENaC activity at the apical membrane and are considered the gold standard for assessing ENaC-mediated Na⁺ transport.

To the best of our knowledge, this is the first study to describe a functional impairment of fetal pulmonary epithelial cells upon acute *Ureaplasma* infection and to reveal potential mechanisms of *Ureaplasma*-driven preterm lung disease. Moreover, this is the first investigation to describe *Ureaplasma*-mediated NH_3_ accumulation as the causative *Ureaplasma* virulence factor. Notably, the present findings might have a significant translational impact. In preterm infants with perinatal alveolar colonization with *Ureaplasma* species, *Ureaplasma*-mediated inhibition of epithelial Na^+^ transport could impair the essential AFC, contribute to lung fluid accumulation, and worsen the respiratory distress of preterm infants. Ultimately, this study underlines the relevance of *Ureaplasma* as true pathogens in preterm infants, especially the most immature ones, and sheds light on the role of urease-inhibiting drugs as potential therapeutic strategies in colonized preterm infants early in life. Urease inhibitors might constitute useful alternatives to antimicrobial strategies in the future. Further studies ought to confirm the observed mechanisms *in vivo* and investigate their role as addressable virulence factors in the clinical context.

## Materials and methods

### Ethics statement

All experimental protocols were approved by the institutional review board (IRB: Landesdirektion Leipzig, Leipzig, Germany, permit number: TVV15/22). All methods were performed in compliance with Directive 2010/63/EU.

### Bacterial strains and culture conditions

*U. parvum* serovar 3 (Up3) and *U. urealyticum* serovar 8 (Uu8), both often associated with disease manifestation [15,56], were obtained from the American Tissue Culture Collection (ATCC, European distributor LGC Standards GmbH, Wesel, Germany; Up3: ATCC 27815, Uu8: ATCC 27618). Frozen stocks were prepared from the mid-logarithmic phase. For each experiment, isolates were inoculated 1:10 with in-house medium containing 82% autoclaved PPLO medium (Becton, Dickinson & Company, Franklin Lakes, NJ, USA), 11% heat-inactivated horse serum (v/v), urea (186 mM, Merck, Taufkirchen, Germany) and 1% phenol red (Merck), and adjusted to pH 6.5 [5,6]. Serial dilutions of both strains were incubated overnight. Determination of color-changing units (CCU) was performed in 96-well plates (Greiner Bio-One, Frickenhausen, Germany) by 10-fold serial dilutions in 200 µl broth [57]. Uu8 and Up3 DNA copy numbers (cn) were determined by qPCR (Table 1). We tested the effects of a 3-hour infection of FDLE cells with *Ureaplasma* to assess potential immediate pathogen-cell interactions and a 24-hour infection to investigate the impact on established cellular and functional responses.

**Table 1:** *Ureaplasma* primer sequences.

Gene	Forward Primer, 5’–3’	Reverse Primer, 3’–5’
Uu8 AF085724.2	CTGACCACGTAGTGGAAGGG	CAACTTGGATAGGACGGTCACC
Up3 AF085732.1	GCTGACCTAATGCAATCTGCTCG	GGACTAAATTGACTTGATGATCCTG

### Isolation and stimulation of FDLE cells

Sprague-Dawley rats were bred at the Medical Research Center of the University of Leipzig. Pregnant rats were euthanized by pentobarbital injection on gestation days E20-21 (term E = 22). A fetal rat lung at E20-21 is in the saccular stage of development, which closely overlaps with the developmental stage of human preterm infants at 24–36 weeks of gestation. Each FDLE cell preparation consisted of two pregnant rats with approximately 30–40 fetal rats of both sexes. The primary rat FDLE cells, a model of preterm ATII cells, were isolated from rat fetal lungs [35]. The fetal lungs were mechanically and enzymatically dissociated, and the resulting cell suspension was subjected to differential centrifugation and selective plating to enrich for epithelial cells with a resulting epithelial purity of > 95%, as previously described [58,59]. The obtained FDLE cells were seeded onto permeable Snapwell inserts (surface area 1.1 cm^2^, Corning, Corning, NY, USA) at a density of 1x10^6^ cells per insert for electrophysiological measurements. For RNA isolation, cells were seeded onto larger inserts (ThinCert, 4.6 cm^2^ surface area, Greiner Bio-One) at a density of 2x10^6^ cells per insert. For all experiments, FDLE cells were cultured under submerged conditions, reproducing the physiological context of the perinatally fluid-filled alveoli. Standard cell culture medium consisted of MEM with 10% fetal bovine serum (FBS, Biochrom, Berlin, Germany), glutamine (2 mM, Life Technologies), and antibiotic-antimycotic (Life Technologies). Four days after isolation, cells were incubated with Uu8 and Up3 cultures corresponding to 10^6^ and 10^8^ CCU/ml viable organisms (corresponding to 1.3x10^6^ – 1.8x10^7^ cn/ml), respectively, diluted 1:5 in cell culture medium for 3 or 24 hours (h), or cultured with NaOH or NH_4_OH (referred to as NH_3_) at pH 8.0 for 24 h. Since NH_3_ exists in rapid pH-dependent equilibrium with NH_4_^+^ in buffered solutions, all exposures represent a mixed NH_3_/NH_4_^+^ system. In the present study, addition of ammonium hydroxide (≈ 13.4 M) to the cell culture medium resulted in a nominal total NH_3_/NH_4_^+^ concentration of ~6.7 mM. At pH 8.0, approximately 5–6% of this pool is present as uncharged NH_3_ (pK ≈ 9.25), corresponding to ~0.35 mM NH_3_. Sealing of the culture plates prevented the medium buffer from neutralizing the pH. The impact of varying *Ureaplasma* inocula was tested in preliminary dose-response experiments using dilutions of 1:5, 1:10, and 1:50 and assessing epithelial Na^+^ transport and transepithelial resistance. Based on maximum effects on Na^+^ transport, minimum impact on epithelial barrier integrity, and *in vivo* amnion fluid *Ureaplasma* concentrations, we used 1:5 dilutions of the given Uu8 and Up3 cultures throughout this study. Finally, co-incubation with flurofamide (10 µM, Merck) was used to inhibit the *Ureaplasma*-specific urease. All experiments were performed with FDLE cell cultures obtained from three independent preparations.

### Ussing chamber analyses

Ussing chamber measurements [60] were performed following a 3- and 24 h incubation with *Ureaplasma*. Only cell monolayers with a *R*_te_ exceeding 300 Ω·cm^2^ were included in the analyses. Electrophysiological solutions consisted of (in mM) 145 Na^+^, 5 K^+^, 1.2 Ca^2+^, 1.2 Mg^2+^, 125 Cl^−^, 25 HCO^3−^, 3.3 H_2_PO_4_^−^ and 0.8 HPO_4_^2−^ (pH 7.4), with 10 glucose (basolateral) or 10 mannitol (apical). The solutions were continuously gassed with carbogen (5% CO_2_, 95% O_2_) during measurements. Equivalent short-circuit currents (*I*_SC_) were determined every 20 s by measuring transepithelial voltage (*V*_te_) and *R*_te_ with a transepithelial current clamp (Physiologic Instruments, San Diego, CA) and calculating the quotient *I*_SC_ = *V*_te_/*R*_te_. After the *I*_SC_ reached a stable plateau (*I*_base_), amiloride (10 µM, Merck) was applied to the apical chamber. The amiloride-sensitive *I*_SC_ (∆*I*_amil_), a measure of ENaC activity, was calculated from the difference between *I*_base_ and *I*_amil_. The apical Na^+^ permeability was determined by adding amphotericin B (100 µM, Merck) to the basolateral compartment. For this setup, 140 mM of basolateral Na^+^ was replaced by 116 mM N-methyl-D-glucamine (NMDG^+^, Merck) and 24 mM choline, generating a 145:5 apical-to-basolateral Na^+^ gradient. The *I*_SC_ was measured every 5 s with a *V*_te_ clamp. Following amphotericin B addition, at *I*_SC_ peak value, *amil*_max_ was determined by apically adding 10 µM amiloride, or ouabain (1 mM, Merck) basolaterally to calculate *ouab*_max_.

### Western blot

Phosphorylation of Erk1/2 (extracellular-signal-regulated kinases) was analyzed using antibodies against phospho-p44/42 MAPK (Erk1/2) at Tyr202/Tyr204 of Erk1 (Thr185 and Tyr187 of Erk2) (#9101, Cell Signaling Technology, Frankfurt am Main, Germany) and Erk1/2 (#9102, Cell Signaling Technology). Furthermore, α-Tubulin (11H10, #2125, Cell Signaling Technology) expression was used as a reference. Suitable secondary antibodies, conjugated to horseradish peroxidase (HRP), were used to detect primary antibodies. HRP activity was analyzed by enhanced chemiluminescence (ECL, Amersham, Piscataway, NJ, USA) on X-ray film. Band intensity was measured by densitometry using Image-J (National Institutes of Health, Bethesda, MD, USA).

### WST-1 assay for cell proliferation and viability

The WST-1 cell proliferation colorimetric assay quantifies cell proliferation, viability, and cytotoxicity by measuring the reduction of water-soluble tetrazolium salt WST-1 to a formazan dye by viable cells and organisms. FDLE cells were seeded in 96-well plates at a density of 2x10^4^ cells per well and infected with *Ureaplasma*. In parallel, viable *Ureaplasma*, derived from the same stock, were equally diluted 1:5 in cell culture media. In addition, uninfected FDLE cells were treated with *Ureaplasma* medium alone, diluted 1:5, serving as negative controls. Then, WST-1 reagent (Roche, Mannheim, Germany) was added to each of the three settings according to the manufacturer’s instructions. Absorbance at 450 nm was measured regularly every 0.5-1 h.

### mRNA expression analyses

RNA isolation was done following a 24-h incubation with *Ureaplasma* using the Purelink RNA Mini Kit (Life Technologies) and DNAse I (Life Technologies) according to the manufacturer’s instructions. For reverse transcription, 1 g of RNA was pre-annealed with Oligo(dT)18 primers (Fisher Scientific GmbH, Schwerte, Germany), followed by the addition of Superscript III (Life Technologies). The Express Greener QPCR Uni-Kit (Fisher Scientific GmbH), gene-specific primers (Table 2), and the CFX 96 Real-Time system (Bio-Rad, Munich, Germany) were used to perform real-time quantitative PCR. Target-specific plasmid DNA was used as the internal standard for absolute quantification. Molecular concentrations were normalized to the mitochondrial ribosomal protein S18a (Mrps18a) reference gene and expressed as the respective control’s relative fold change (RFC).

**Table 2:** Primer sequences.

Gene	Forward Primer, 5’–3’	Reverse Primer, 3’–5’
*α-ENaC* NM031548.2	TTCTGGGCGGTGCTGTGGCT	GCGTCTGCTCCGTGATGCGG
*β-ENaC* NM012648.1	TGCAGGCCCAATGCCGAGGT	GGGCTCTGTGCCCTGGCTCT
*γ-ENaC* NM017046.1	CACGCCAGCCGTGACCCTTC	CTCGGGACACCACGATGCGG
*Na,K-ATPases- α*_*1*_ NM012504.1	GGACGAGACAAGTATGAGCCCGC	CATGGAGAAGCCACCGAACAGC
*Na-K-ATPases- β*_*1*_ NM012505.2	GCGCAGCACTCGCTTTCCCT	GGGCCACACGGTCCTGGTACG
*Sftpa* NM001270645.1	CCTCTTCTTGACTGTTGTCGCTGG	GCTGAGGACTCCCATTGTTTGCAG
*Sftpb* NM138842.1	GGAGCTAATGACCTGTGCCAAGAG	CTGGCCCTGGAAGTAGTCGATAAC
*Sftpc* NM017342.2	GATGGAGAGCCCACCGGATTACTC	GAACGATGCCAGTGGAGCCAATAG
*Mrps18a* NM198756.1	GCGACCGGCTGGTTATGGCT	GGGCACTGGCCTGAGGGATTAG

### Statistical analyses

Statistical analyses were performed using GraphPad Prism software (GraphPad Prism 9.1.1, https://www.graphpad.com, GraphPad Software, La Jolla, CA, USA). Data are presented as means ± standard deviation (SD) and listed in S1 Table. Statistical significance was defined as *p*-value < 0.05.

## Supporting information

S1 Fig Detection of pErk1/2 and Erk1/2 in FDLE cells by Western blot analysis. Original blots of Fig 4.
(DOCX)

S2 Fig Detection of pErk1/2 and Erk1/2 in FDLE cells by Western blot analysis. Original blots of Fig 7A.
(DOCX)

S3 Fig Detection of pErk1/2 and Erk1/2 in FDLE cells by Western blot analysis. Original blots of Fig 9A.
(DOCX)

S1 Table Mean, SD and *p*-values of data. (DOCX)
