# Supplementary material for: Ureaplasma-driven inhibition of the epithelial Na+ transport in fetal alveolar cells: A novel mechanism of Ureaplasma-mediated preterm lung disease
Source: PLoS Pathog. 2025 Dec 29;21(12):e1013837. doi: 10.1371/journal.ppat.1013837 (PMC12768415; doi:10.1371/journal.ppat.1013837)
Supplement: S2 Fig — Original blots of Fig 7A. (DOCX) [file ppat.1013837.s002.docx]

***Ureaplasma*-driven inhibition of the** **epithelial Na^+^ transport in fetal alveolar cells: a novel mechanism of *Ureaplasma*-mediated preterm lung disease**

Kirsten Glaser, Carl-Bernd Rieger, Elisabeth Paluszkiewicz, Ulrich H. Thome, Mandy Laube

**S2 Fig**

Detection of pErk1/2 and Erk1/2 in FDLE cells by Western blot analysis. Detection of a-tubulin served as a loading control. Original blots of Fig 7A.

| **pErk1/2**  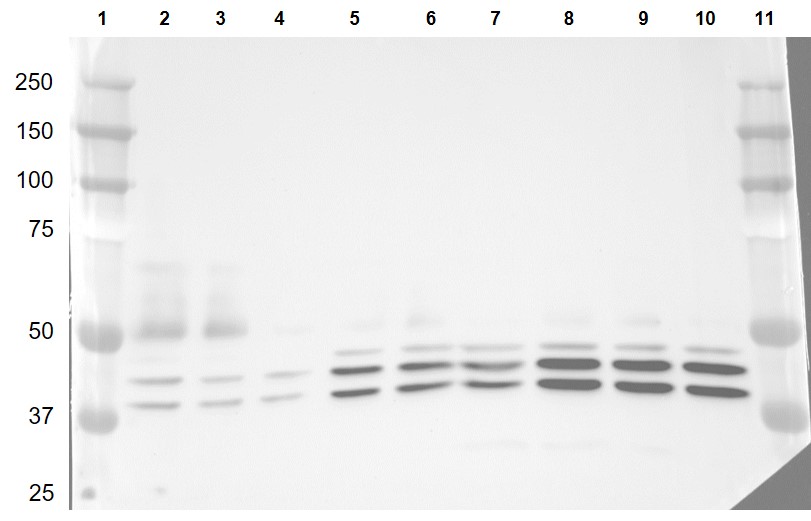 | Samples   1. Marker; MW as indicated at the left (kDa) 2. Control #1 3. Control #2 4. Control #3 5. NaOH #1 6. NaOH #2 7. NaOH #3 8. NH_3_ #1 9. NH_3_ #2 10. NH_3_ #3 11. Marker |
| --- | --- |
| **Erk1/2**  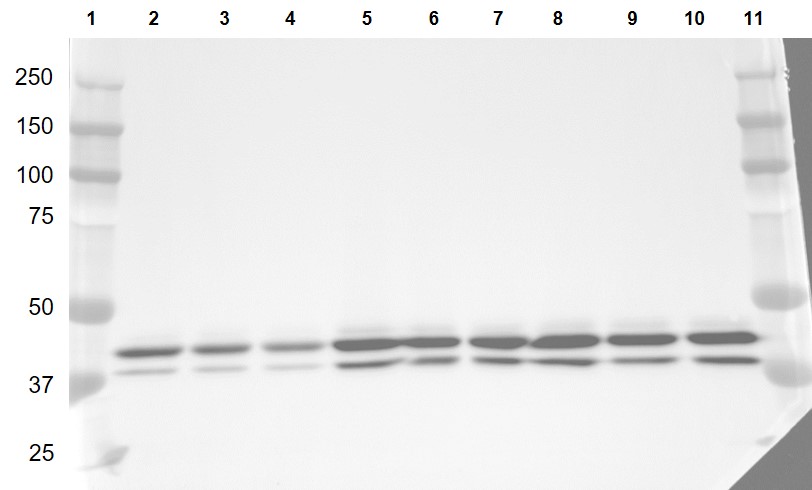 |  |
| **a-tubulin**  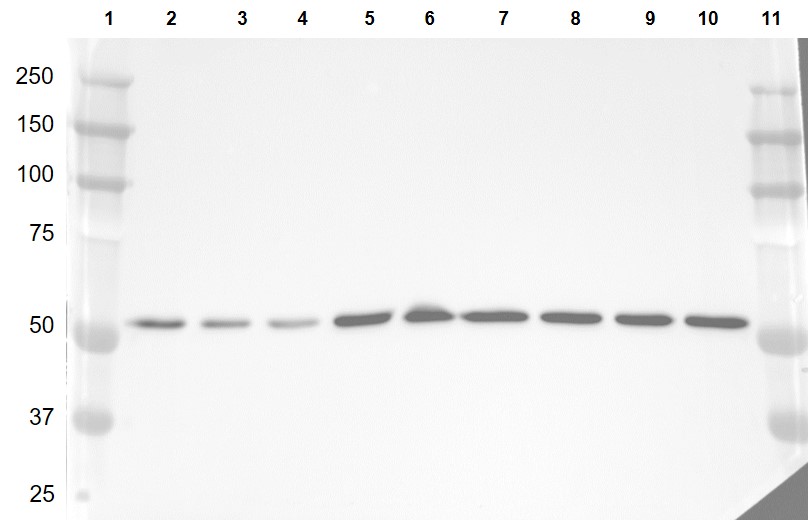 |  |
