# Supplementary material for: Ureaplasma-driven inhibition of the epithelial Na+ transport in fetal alveolar cells: A novel mechanism of Ureaplasma-mediated preterm lung disease
Source: PLoS Pathog. 2025 Dec 29;21(12):e1013837. doi: 10.1371/journal.ppat.1013837 (PMC12768415; doi:10.1371/journal.ppat.1013837)
Supplement: S3 Fig — Original blots of Fig 9A. (DOCX) [file ppat.1013837.s003.docx]

***Ureaplasma*-driven inhibition of the** **epithelial Na^+^ transport in fetal alveolar cells: a novel mechanism of *Ureaplasma*-mediated preterm lung disease**

Kirsten Glaser, Carl-Bernd Rieger, Elisabeth Paluszkiewicz, Ulrich H. Thome, Mandy Laube

**S3 Fig**

Detection of pErk1/2 and Erk1/2 in FDLE cells by Western blot analysis. Detection of a-tubulin served as a loading control. Original blots of Fig 9A.

| **pErk1/2**  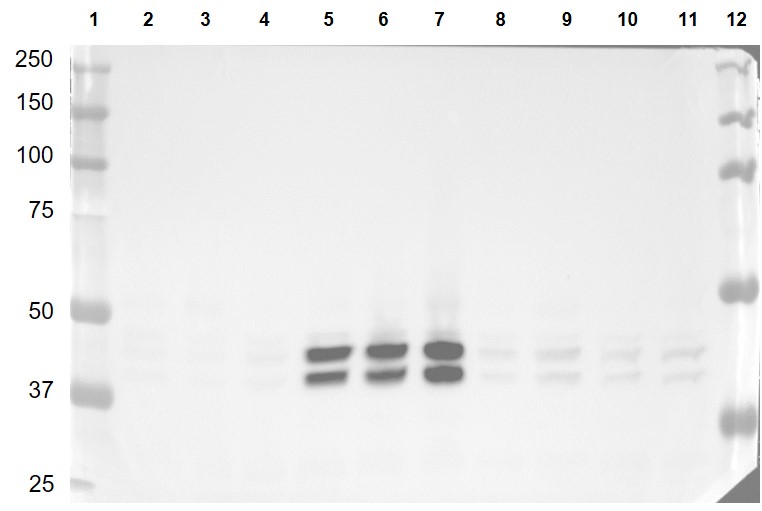 | Samples   1. Marker; MW as indicated at the left (kDa) 2. Control #1 3. Control #2 4. Control #3 5. Uu8-infected #1 6. Uu8-infected #2 7. Uu8-infected #3 8. Uu8-infected/flurofamide #1 9. Uu8-infected/flurofamide #2 10. Uu8-infected/flurofamide #3 11. Uu8-infected/flurofamide #4 12. Marker |
| --- | --- |
| **Erk1/2**  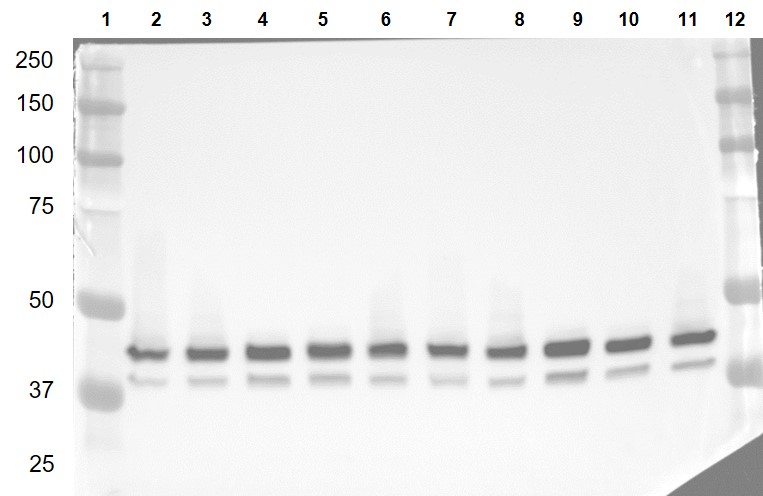 |  |
| **a-tubulin**  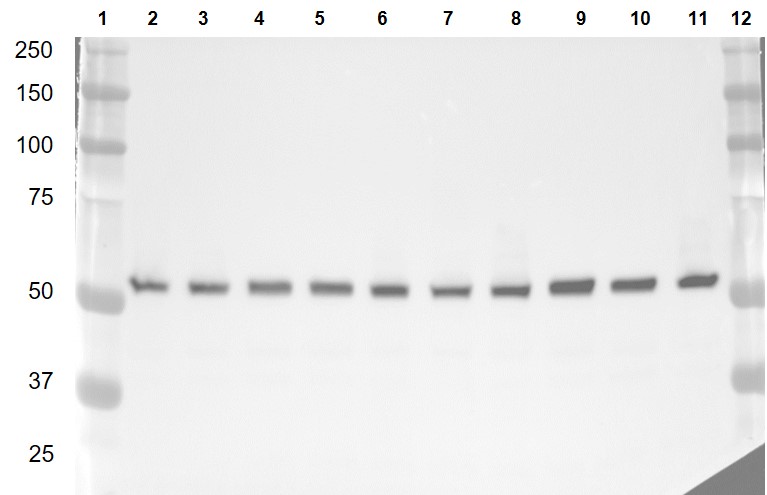 |  |
