# Supplementary material for: Ureaplasma-driven inhibition of the epithelial Na+ transport in fetal alveolar cells: A novel mechanism of Ureaplasma-mediated preterm lung disease
Source: PLoS Pathog. 2025 Dec 29;21(12):e1013837. doi: 10.1371/journal.ppat.1013837 (PMC12768415; doi:10.1371/journal.ppat.1013837)
Supplement: S1 Table — (DOCX) [file ppat.1013837.s004.docx]

***Ureaplasma*-driven inhibition of the** **epithelial Na^+^ transport in fetal alveolar cells: a novel mechanism of *Ureaplasma*-mediated preterm lung disease**

Kirsten Glaser, Carl-Bernd Rieger, Elisabeth Paluszkiewicz, Ulrich H. Thome, Mandy Laube
